# Inhibition of CYR61-S100A4 Axis Limits Breast Cancer Invasion

**DOI:** 10.3389/fonc.2019.01074

**Published:** 2019-10-23

**Authors:** Johanna W. Hellinger, Silke Hüchel, Lena Goetz, Gerd Bauerschmitz, Günter Emons, Carsten Gründker

**Affiliations:** Department of Gynecology and Obstetrics, University Medicine Göttingen, Göttingen, Germany

**Keywords:** breast cancer, CYR61, invasion, EMT, triple negative breast cancer

## Abstract

**Background and Objective:** Matricellular proteins modulate the micro environment of tumors and are recognized to contribute to tumor cell invasion and dissemination. The cysteine-rich angiogenic inducer 61 (CYR61) is upregulated in mesenchymal transformed and invasive breast cancer cells. CYR61 correlates with poor prognosis of breast cancer patients. The signaling mechanism that causes invasive properties of cancer cells regarding to epithelial-mesenchymal transition (EMT) needs further research. In this study, we investigated the signaling mechanism, which is responsible for reduced cell invasion after suppression of CYR61 in mesenchymal transformed breast cancer cells and in triple negative breast cancer cells.

**Methods:** We addressed this issue by generating a mesenchymal transformed breast cancer cell line using prolonged mammosphere cultivation. Western blotting and quantitative PCR were used to analyze gene expression alterations. Transient gene silencing was conducted using RNA interference. Proliferation was assessed using AlamarBlue assay. Invasiveness was analyzed using 2D and 3D invasion assays. Immune-histochemical analysis of patient tissue samples was performed to examine the prognostic value of CYR61 expression.

**Results:** In this study, we investigated whether CYR61 could be used as therapeutic target and prognostic marker for invasive breast cancer. We discovered an interaction of CYR61 with metastasis-associated protein S100A4. Suppression of CYR61 by RNA interference reduced the expression of S100A4 dependent on ERK1/2 activity regulation. Non-invasive breast cancer cells became invasive due to extracellular CYR61 supplement. Immune-histochemical analysis of 239 patient tissue samples revealed a correlation of higher CYR61 and S100A4 expression with invasive breast cancer and metastasis.

**Conclusion:** Our data suggest that suppression of CYR61 impedes the formation of an invasive cancer cell phenotype by reducing ERK1/2 phosphorylation thereby suppressing S100A4. These findings identify mechanisms by which CYR61 suppresses cell invasion and suggest it to be a potential therapeutic target and prognostic marker for invasive breast cancer and metastasis.

**Graphical Abstract:**
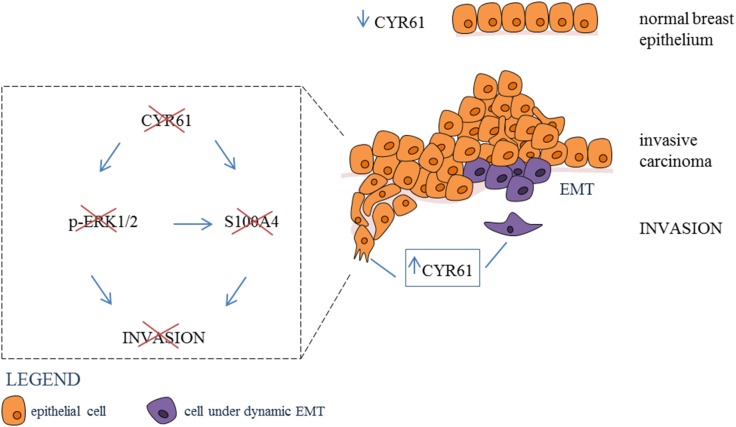
Inhibtion of CYR61-S100A4 axis limitis breast cancer Invasion. CYR61 expression is low in normal breast epithelium, while expression is increased in invasive breast carcinoma. Suppression of CYR61 leads to reduced ERK1/2 phosphorylation and S100A4 expression thereby reducing breast cancer invasion. EMT, epithelial-mesenchymal transition.

## Introduction

In 2019, approximately 271270 woman and men in the United States will be diagnosed with breast cancer. Due to improved early detection techniques and treatment options 5-year-survival rates for local and regional breast cancer are 84–99 %. However, only 27of patients diagnosed with distant metastasis survive a period of 5 years (1). Consequently it is necessary to identify prognostic markers for the early detection of breast cancer metastasis and new treatment options for this indications which accounts for more than 90% of cancer related death (2).

The first key event in the multi-step process of metastasis is the separation of tumor cells from the primary tumor and the dissemination into the surrounding tissue. Cells gain the ability to migrate and invade by altering their cytoskeletal organization, cell-cell-contacts, contacts with the extracellular matrix (ECM) and surrounding stroma (3). Epithelial-mesenchymal transition (EMT) is a transient dynamic program induced by different transcription factors (TFs). EMT-TFs orchestrate tumor-promoting micro environmental changes, cancer cell stemness, and chemo resistance (4, 5). The contribution of EMT programs to the metastatic cascade regarding breast cancer is supported by several publications (6–8). However, it is still under debate if an involvement of EMT programs is indispensable for creating an invasive phenotype (4). Therefore it is necessary to study cancer cell invasion with regards to EMT complexity (9, 10).

The cysteine rich angiogenic inducer (CYR61) belongs to the CCN family (CYR61, CTGF /CCN12, NOV/CCN3, WISP-1/CCN4, WISP-2/CCN5, WISP-3/CCN6) of matricellular proteins and is localized on cell surface, cytoplasm and as a secreted protein in the extracellular matrix. The functions of CYR61 are cell type and context-dependent (11). They are transmitted through binding to integrin and heparin sulfate proteoglycans (HSPGs). CYR61 was shown to be involved in facilitating EMT programs in different cancer entities (12–14). It is known that elevated CYR61 expression promotes tumor progression, proliferation, migration and invasion of breast cancer (15, 16), whereas the role of CYR61 in breast cancer EMT programs remains elusive. Otherwise, CYR61 can act as a tumor suppressor in non-small cell lung cancer (17) and in fibroblasts by inducing apoptosis and senescence during wound healing (18, 19). The role of CYR61 signaling in cancer invasion and EMT programs regarding to a potential use as therapeutic target and prognostic marker needs further evaluation.

We hypothesize that CYR61 is a key regulator of breast cancer invasion. We want to identify the mechanisms by which CYR61 facilitates an invasive phenotype. Furthermore, we want to investigate the value of CYR61 as a therapeutic target and prognostic marker for invasive and metastatic breast cancer.

## Materials and Methods

### Cell Lines and Cell Culture

Human breast cancer cell lines MCF-7, T47D, MDA-MB-231, and HCC1806 were obtained from American Type Cell Collection (ATCC; Manassas, VA, USA) and cultured in minimum essential medium (MEM; biowest, Nuaillé, France) supplemented with 10% fetal bovine serum (FBS; biochrom, Berlin, Germany), 1% Penicillin/Streptomycin (P/S; Gibco, Carlsbad, CA, USA), 0,1% Transferrin (Sigma, St. Louis, USA) and 26 IU Insulin (Sanofi, Frankfurt, Germany). Human osteosarcoma cell line MG-63 was purchased from ATCC and cultured Dulbecco's modified eagle medium (DMEM; Gibco) supplemented with 10% FBS (biochrom) and 1% Penicillin/Streptomycin (Gibco). To retain identity of cell lines, purchased cells were expanded and aliquots were frozen in liquid nitrogen. A new frozen stock was used every half year and Mycoplasma testing of cultured cell lines was performed routinely using PCR Mycoplasma Test Kit I/C (PromoCell GmbH, Heidelberg, Germany). All cells were cultured in a humidified atmosphere with 5% CO_2_ at 37°C.

### Generation of Mesenchymal Transformed MCF-7 Cells

Mesenchymal transformed MCF-7 breast cancer cells (MCF-7-EMT) were generated as described earlier (20). Briefly, 4 × 10^4^ cells/ml were cultured in prolonged mammosphere culture (5–6 weeks) in ultralow adherence six well plates (Corning, Lowell, MA, USA) in DMEM/F12 (Gibco) supplemented with 10% charcoal-stripped fetal calf serum (cs-FCS;PAN-biotech, Aiden Bach, Germany), 2% B27 supplement (Invitrogen, Darmstadt, Germany), 1% penicillin/streptomycin, 0.5 mg/ml hydrocortisone (Sigma, St. Louis, MO, USA), 5 μg/ml insulin, 20 ng/ml epidermal growth factor (EGF; Sigma, St. Louis, MO, USA).

### Treatment With rhCYR61 and U0126

Human breast cancer cells were seeded at 5 × 10^5^ cells/ml in MEM supplemented with 10% FB, 1% P/S, 0,1% Transferrin 26 IU Insulin. Cells treated with 1 μg/ml rhCYR61 (recombinant human CYR61; C-63398; PromoKine; Heidelberg; Germany) were serum-deprived 24 h prior to treatment and lysed 24 h after treatment. Cells treated with 10 μM U0126 (#tlrl-u0126; InvivoGen; San Diego; USA) were lysed 24 h after treatment.

### Transwell Invasion Assay

Using co-culture transwell assay as described earlier (21), 1 × 10^4^ breast cancer cells were seeded in DMEM w/o phenol red (Gibco), supplemented with 10% cs-FCS into a cell cultural insert (upper well) with a polycarbonate membrane (8 μm pore diameter, Merck Millipore, Cork, Ireland) coated with 30 μL of a Matrigel^®^ (BD Bioscience, Bedford, MA, USA) solution (1:2 in serum-free DMEM). The osteosarcoma cells were seeded (2.5 × 10^4^) in DMEM supplemented with 10% cs-FCS into the lower well (24-well-plate). After 24 h cells were co-cultured for 48 h or 96 h. Stably transfected cells (overexpressing CYR61 or S100A4) were seeded at a density of 1 × 10^4^ per well in DMEM w/o phenol red cell cultural insert (upper well, Matrigel-coated with a polycarbonate membrane), with the lower well containing DMEM w/o phenol red supplemented with 10% cs-FBS and cultured for 96 h. Invaded cells on the lower side of the insert were stained with hematoxylin and the number of cells in four randomly selected fields of each insert was counted.

### 3D Spheroid Assay

Assessment of 3D cell invasion was pursued as describes earlier with minor changes (22). Briefly 1 × 10^3^ breast cancer cells were seeded in 100 μL in a well of an ultra-low-adherence 96-well plate (ULA; Nexcelom, Cenibra GmbH, Bramsche, Germany). After 48 h spheroid formation was visually confirmed and 50 μL of media was removed. Thereafter 50 μL Matrigel were added to the spheroid wells. Central position of the spheroids was checked visually and Matrigel was allowed to solidify for 1 h at 37°C and 5% CO_2_. Afterwards 50 μL media were added to each well and a picture was taken marking time point 0 (t0h). When indicated 1μg/ml rhCYR61 or 10μM U0126 were added. Spheroid growth area was analyzed using ImageJ polygonal selection and measurement. Mean values were calculated and compared to respective control.

### Small Interfering RNA Transfection

Breast cancer cell lines MCF-7-EMT (5 × 10^5^ cells/ml) and MDA-MB-231 (2.5 × 10^5^ cells/ml) were seeded in 2 ml of MEM with 10% FBS (-P/S) in 25 cm^2^ cell culture flask. The cells were transiently transfected with siRNA specific to S100A4 (sc-106781 pool of three S100A4-specific siRNAs; Santa Cruz Biotechnology, Dallas, USA), CYR61 (sc-39331 pool of three CYR61-specific siRNAs; Santa Cruz Biotechnology) or YAP1 (sc38637 pool of three YAP1 specific siRNAs; Santa Cruz Biotechnologies) in OPTI-MEM I medium (Gibco, Carlsbad, CA, USA) with siRNA transfection reagent (sc-29528; Santa Cruz Biotechnology, Dallas, USA). A non-targeting siRNA was used as control (sc-37007 control-A; Santa Cruz Biotechnology, Dallas, TX, USA). After an incubation period of 6 h, MEM supplemented with 20% FBS and 20% penicillin/streptomycin was added.

### Immune-Histochemical Staining

Immune-histochemical staining of human tissue array slides (T087a; BR20837; BR248a; US Biomax, Derwood, MD, USA) was performed as described earlier (23). Sample sections were deparaffinized and rehydrated. Then antigens were retrieved by slide incubation in 0.01 M citrate buffer (pH 6.0) in microwave (700W) for 5 min. Using 3% hydrogen peroxidase solution for 6 min the endogenous peroxidase activity was quenched. Sample sections were incubated over night with primary labeled antibodies against S100A4 (NBP2-54580AF488; Alexa Fluor 488 labeled; 5 μg/ml; Novus Biologicals, Centennial, CO, USA) and CYR61 (NB100-356R; DyLight labeled; 5 μg/ml; Novus Biologicals) at 4°C. Staining was visualized using a Zeiss Scope A1 Axio microscope (ZEISS, Oberkochen, Germany) with an oil EC PLAN-NEOFLUAR 100x (ZEISS, Oberkochen, Germany) objective and the ZEN software (ZEISS, Oberkochen, Germany).

### Real-Time Quantitative PCR Analysis

Total RNA was extracted using an RNeasy mini kit (Qiagen, Hilden, Germany) and 2 μg were reverse transcribed with high capacity cDNA reverse transcription kit (Qiagen, Hilden, Germany). Real- time qPCR was performed using SYBR green PCR master mix kit (Qiagen, Hilden, Germany). Primer were, for S100A4 5′- GTACTCGGGCAAAGAGGGTG−3′ (forward) 5′- TTGTCCCTGTTGCTGTCCAA−3′ (reverse), for CYR61 5′- CTCCCTGTTTTTGGAATGGA−3′ (forward) 5′- TGGTCTTGCTGCATTTCTTG−3′ (reverse), for YAP1 5′- TCCCAGATGAACGTCACAGC−3′ (forward) 5′- TCATGGCAAAACGAGGGTCA−3′ (reverse), E-cadherin 5′-CCTCCTGAAAAGAGAGTGGA−3′ (forward) 5′-GTGTCCGGATTAATCTCCAG−3′ (reverse), Vimentin 5′-GCTGCTAACTACCAAGACAC−3′ (forward) 5′-TCAGGTTCAGGGAGGAAAAG−3′ (reverse), Zeb1 5′-AAGACAAACTGCATATTGTGGAAG−3′ (forward) 5′-CTGCTTCATCTGCCTGAGCTT−3′ (reverse), SNAI1 5′-GCCAAACTACAGCGAACTGG−3′ (forward) 5′- GAGAGAGGCCATTGGGTAGC-3′ (reverse), SNAI2 5′- AAGATGCACATCCGAAGCCA-3′ (forward) 5′- CATTCGGGAGAAGGTCCGAG−3′ (reverse) and GAPDH 5′- GAAGGTCGGAGTCAACGGAT−3′ (forward) 5′- TGGAATTTGCCATGGGTGGA−3′ (reverse). PCR conditions were: denaturing once at 95°C (2 min), 95°C (5 s), and 60°C (15 s) for 40 cycles.

### Western Blot Analysis

Cells were lysed in cell lytic M buffer (Sigma, St. Louis, USA) supplemented with 0.1% phosphatase-inhibitor (Sigma, St. Louis, MO, USA) and 0.1% protease-inhibitor (Sigma, St. Louis, MO, USA). Isolated proteins (40 μg) were fractioned using 12% SDS gels and electro-transferred to a polyvinylidene difluoride membrane (Merck Millipore, Cork, Ireland). Primary antibodies against S100A4 1:250 (HPA007973; Sigma, St. Louis, USA), CYR61 1:250 (HPA029853; Sigma, St. Louis, MO, USA), YAP 1:250 (sc-398182; Santa Cruz Biotechnology, Dallas, TX, USA), ERK1/2 1:1000 (4695S;Cell Signaling Technologies Inc., Danvers, MA, USA), Phospho-ERK1/2(Thr202/Tyr204) 1:1000 (9101S; Cell Signaling Technologies Inc.), and GAPDH 1:2000 (5174; Cell Signaling Technologies Inc) were used. The membrane was washed and incubated in horseradish peroxidase-conjugated secondary antibodies (GE Healthcare, Buckinghamshire, UK). Antibody-bond protein bands were assayed using a chemiluminescent luminol enhancer solution (Cyanagen, Bologna, Italy).

### ECM Degradation

Wells of a 96-well plate were coated at room temperature for 20 min with 0.05 mg/ml Poly-L-lysine in DPBS (Sigma) and 15 min with glutaraldehyde 0.5% in DPBS. Gelatin (2 mg/ml; G9391; Sigma) was FITC conjugated as recommend by manufacture (#343210; EMD Millipore Corp., Billeria, MA, USA). Wells were coated with 60 μL FITC-conjugated gelatin (2 mg/ml; Invitrogen, Milpitas, CA, USA) diluted 1:5 with unlabeled gelatin (Sigma) and incubated for 10 min at RT. Solution was discarded and wells were incubated for 30 min in 70% ethanol and afterwards free aldehydes were quenched with culture media for 30 min at room temperature before cells were seeded. Cells were seeded (4.4 × 10^3^ cells per Well) and treated with rhCTGF (1 μg/ml; R&D systems). After 24 h proteolytic activity was detected by measuring fluorescence (extinction 490 nm/emission 520 nm) using Synergy (BioTek Instruments, Bad Friedrichshall, Germany). Each experiment was performed in duplicates for at least three times. Mean values were compared to the respective control.

### AlamarBlue Assay

3D spheroids were grown as described above and 48 h after adding Matrigel AlamarBlue (BioRad, Hercules, USA) was added and incubated for 4 h at 37°C 5% CO_2_. The colorimetric change of resazurin to resorufin upon cellular metabolic reduction was measured by absorbance reading at 540 nm and 630 nm, using Synergy (BioTek Instruments). Relative AlamarBlue Reduction was calculated as indicated by manufacturer.

### KM Plotter Analysis

The database of the Kaplan-Meier plotter (www.kmplot.com) downloads information of gene expression and overall survival from Gene Expression Omnibus (GEO; only Affymetrix microarrays), the European Genome- Phenome Archive (EGA) and The Cancer Genome Atlas (TCGA). To be able to analyze the prognostic value (overall survival) of CYR61 in 1,402 patient samples, the samples were split into two cohorts according to the expression of quantiles of CYR61 where all possible cutoff values between the lower and the upper quantiles are computed and the best performing threshold is used as a cutoff. These groups are compared by a Kaplan-Meier survival plot and the hazard ratio with 95% confidence intervals. Redundant samples were removed, biased arrays excluded and the proportional hazard assumption was set to zero (24).

### Statistical Analysis

All experiments were performed at least in three biological and technical replicates. Data were analyzed by GraphPad Prism (GraphPad software Inc., v. 7.03, La Jolla, Ca, USA) using unpaired, two-tailed, parametric *t*-test comparing two groups (treatment to respective control) by assuming both populations have the same standard derivation. *P* < 0.05 was considered statistically significant.

## Results

### CYR61 Expression Correlates With Altered Breast Cancer Cell Invasion

**Figure 1:**
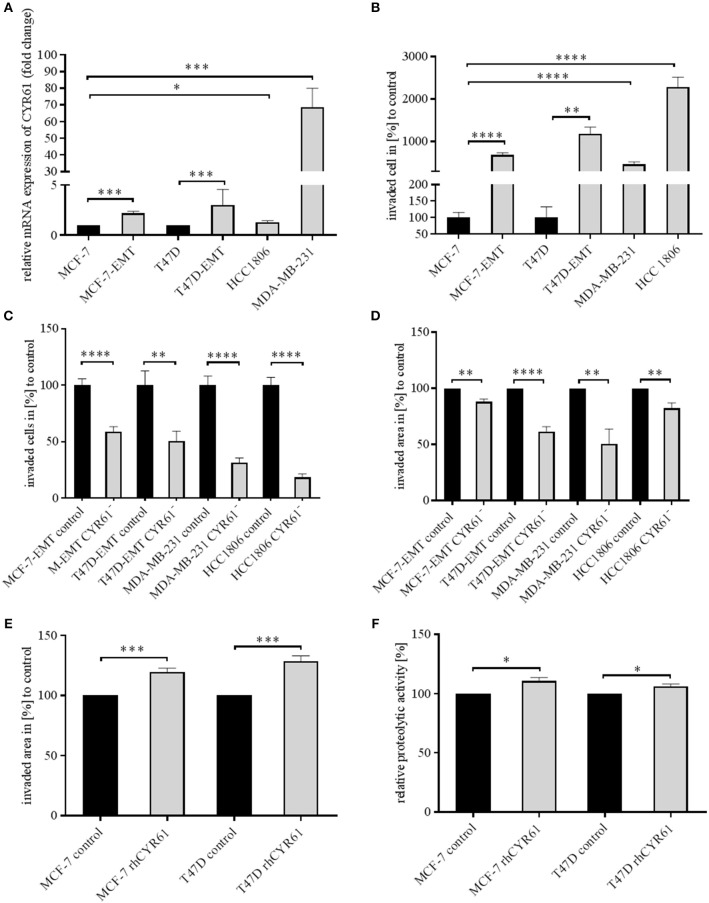
CYR61 expression correlates with breast cancer cell invasiveness. **(A)** Relative CYR61 expression of invasive breast cancer cell lines compared to non-invasive controls. Data represent mean ± SEM. Using unpaired, two-tailed *t*-test analysis. MCF-7-EMT *n* = 5; T47D-EMT *n* = 6; MDA-MB-231 *n* = 4; HCC1806 *n* = 3; ^*^P < 0.05*;* ^***^P < 0.005 **(B)** 2D Invasion analysis of co-cultured (MG-63, osteosarcoma cells) breast cancer cells for 96 h. Percentage of cell invasion compared to controls was assessed by counting invaded cells under the filter in 4 random filter regions. Data represent mean ± SEM. Using unpaired, two-tailed *t*-test analysis. MCF-7-EMT *n* = 36; T47D-EMT *n* = 36; MDA-MB-231 *n* = 24; HCC1806 *n* = 54; ^**^P < *0.01;* ^****^P < 0.0001 **(C)** 2D Invasion analysis of co-cultured (MG-63, osteosarcoma cells) breast cancer cells transient transfected with CYR61 siRNA for 96 h. Percentage of cell invasion compared to controls was assessed by counting invaded cells under the filter in 4 random filter regions. Data represent mean ± SEM. MCF-7-EMT *n* = 36; T47D-EMT *n* = 18; MDA-MB-231 *n* = 18; HCC1806 *n* = 36; ^**^P < 0.01*;* ^****^P < 0.0001 **(D)** 3D invasion analysis of breast cancer spheroids seeded after transient siRNA transfection. Spheroid area was assessed 48 h after adding Matrigel using polygonal selection and compared to spheroid area at time point 0 (adding of Matrigel). Area growth was compared to area growth of control spheroids. Data represent mean ± SEM. Using unpaired, two-tailed *t*-test analysis. MCF-7-EMT *n* = 5; T47D-EMT *n* = 6; MDA-MB-231 *n* = 5; HCC1806 *n* = 6; ^**^P < 0.01*;* ^***^P < 0.005 **(E)** 3D invasion analysis of breast cancer spheroids treated with recombinant human CYR61 (rhCYR61). Spheroid area was assessed 48 h after adding Matrigel and rhCYR61 using polygonal selection and compared to spheroid area at time point 0 (adding of Matrigel+ rhCYR61). Area growth was compared to area growth of control spheroids. Data represent mean ± SEM. Using unpaired, two-tailed *t*-test analysis. MCF-7 *n* = 3; T47D *n* = 3; ^***^ *P* < 0.005 **(F)** Proteolytic activity of non-invasive breast cancer cells treated with rhCYR61 was asses by measurement of fluorescence 24 h after seeding cells on wells coated with FITC-labeled gelatin. Relative proteolytic activity of rhCYR61 treated cells was compared to proteolytic activity of control cells. Data represent mean ± SEM. Using unpaired, two-tailed *t*-test analysis. MCF-7 *n* = 3; T47D *n* = 3 ^*^P < 0.05.

Mesenchymal transformed breast cancer cells show a TGFβ-dependent increased invasive and metastatic potential (20). Despite, it is still under debate, if EMT programs are indispensable for cell invasion (4) and which key players are crucial for pathological EMT programs. We investigated whether within dynamic EMT programs or triple-negative breast cancer (TNBC; no expression of estrogen or progesterone and no overexpression of Her2neu) cells show changes in CYR61 expression. It was shown before that non-invasive breast cancer cells gain invasiveness when co-cultured with primary osteoblasts or osteosarcoma cells (21). Gründker et al. suggested that mesenchymal transformed non-invasive MCF-7 cells (MCF-7-EMT) show an increased invasiveness and elevated CYR61 expression (23). Increased invasiveness could be suppressed by reducing extracellular CYR61 using blocking antibodies. Despite, it remains elusive, if targeting intracellular CYR61 alters cell invasiveness in 2D transwell co-culture invasion assay. Two non-invasive estrogen positive cell lines (MCF-7, T47D) were mesenchymal transformed (MCF-7-EMT; T47D-EMT) and altered expression of EMT-Transcriptionfactors (EMT-TFs) was assessed. Mesenchymal transformation using prolonged mammosphere culture leads to a decreased E-cadherin expression (Figure 1, Figure S1B) in two different estrogen positive breast cancer cells lines. Transforming growth factor induced (TGFBI), Zinc Finger E-Box Binding Homeobox 1 (Zeb1) and Snail Family Transcriptional Repressor 2 (Snai2) expression was increased after mesenchymal transformation (Figure 1, Figures S1A,D–F), while vimentin expression was upregulated in MCF-7-EMT breast cancer cells (Figure 1, Figure S1C) and Snail Family Transcriptional Repressor 1 (Snai1) was upregulated in T47D-EMT cells. In addition CYR61 expression is upregulated in mesenchymal transformed breast cancer cells (Figure 1A; MCF-7-EMT: 2.18 ± 0.2 SEM relative expression compared to MCF-7; *n* = 5; T47D-EMT: 3.04 ± 0.62 SEM relative expression compared to T47D) and in TNBC cells (Figure 1A; MDA-MB-231: 68.67±11.27 SEM relative expression compared to MCF-7; *n* = 4; HCC1806: 1.3 ± 0.09 SEM relative expression compared to MCF-7; *n* = 3). Moreover, mesenchymal transformed and TNBC cell lines show increased invasiveness in a 2D transwell co-culture invasion assay (Figure 1B; MCF-7-EMT: 683.9 ± 53.25 SEM invaded cells in % to MCF-7; *n* = 36; *P* < 0.0001; T47D-EMT: 11881 ± 155.8 SEM invaded cells in % to T47D; *n* = 36; *P* = 0.0022; MDA-MB-231: 466.7 ± 58.52 SEM invaded cells in % to MCF-7; *n* = 24; *P* < 0.0001; HCC1806: 2277 ± 237.4 SEM invaded cells in % to MCF-7; *n* = 54; *P* < 0.0001). To determine whether intracellular suppressed CYR61 regulates breast cancer cell invasion, we transiently reduced CYR61 (see verification of CYR61 suppression Figure 1, Figure S2A) in different invasive breast cancer cell lines and analyzed invasiveness using 2D invasion co-culture assay. Reducing CYR61 results in decreases cell invasion (Figure 1C; MCF-7-EMT CYR61^−^: 59.01 ± 4.34 SEM invaded cells in % to MCF-7-EMT control; *n* = 36; *P* < 0.0001; T47D-EMT CYR61^−^: 50.73 ± 8.71 SEM invaded cells in % to T47D-EMT control; *n* = 36; *P* = 0.002; MDA-MB-231 CYR61^−^: 31.44 ± 4.22 SEM invaded cells in % to MDA-MB-231 control; *n* = 18; *P* < 0.0001; HCC1806 CYR61^−^:18.51 ± 2.96; *n* = 18; *P* < 0.0001). To confirm the impact of CYR61 suppression on breast cancer cell invasion, we assessed whether CYR61 suppression leads to a reduced 3D spheroid invasion growth. Reducing CYR61 results in a decreased 3D spheroid invasion area (Figure 1D; MCF-7-EMT CYR61^−^: 87.93 ± 2.54 SEM invaded area in % to MCF-7-EMT control; *n* = 5; *P* = 0.0014; T47D-EMT CYR61^−^: 61.56 ± 4.3 SEM invaded area in % to T47D-EMT control; *n* = 6; *P* < 0.0001; MDA-MB-231 CYR61^−^:50.37 ± 13.29; *n* = 5; *P* = 0.006; HCC1806 CYR61^−^:82.24 ± 4.81 SEM invaded area in % to HCC1806 control; *n* = 6; *P* = 0.004). To determine whether decreased 3D spheroid invaded area is due to altered proliferation AlamarBlue Assay was conducted. Transient reduces CYR61 does not alter proliferation in 3D breast cancer cell spheroids after 96 h (Figure 1, Figure S2B). Furthermore, increased extracellular CYR61 expression increases 3D spheroid invaded area of non-invasive estrogen positive breast cancer cells (Figure 1E; MCF-7 rhCYR61: 119.7 ± 2.93 SEM invaded area in % to MCF-7 control; *n* = 5; *P* = 0.001; T47D rhCYR61: 128.6 ± 4.38 SEM invaded area in % to T47D control; *n* = 4; *P* = 0.0006). The underlying mechanism of cell invasion into the surrounding tissue evolve different processes including altered cell-cell adhesion, cell-ECM adhesion and ECM degradation (3). Proteolytic activity of estrogen positive breast cancer cells treated with extracellular CYR61 was increased (Figure 1F; MCF-7 rhCYR61: 110.8 ± 2.65 SEM relative proteolytic activity in % compared to MCF-7 control; *n* = 3; *P* = 0.015; T47D rhCYR61: 106.2 ± 1.806 SEM relative proteolytic activity compared to T47D control; *n* = 3; *P* = 0.026), while proliferation was not altered (Figure 1, Figure S2C). Collectively, these data indicate that suppression CYR61 decreases invasiveness in mesenchymal transformed and TNBC cells. Furthermore, increased extracellular CYR61 expression increases invasiveness of non-invasive estrogen positive breast cancer cells.

### Suppression of CYR61 Reduces S100A4 Expression

**Figure 2:**
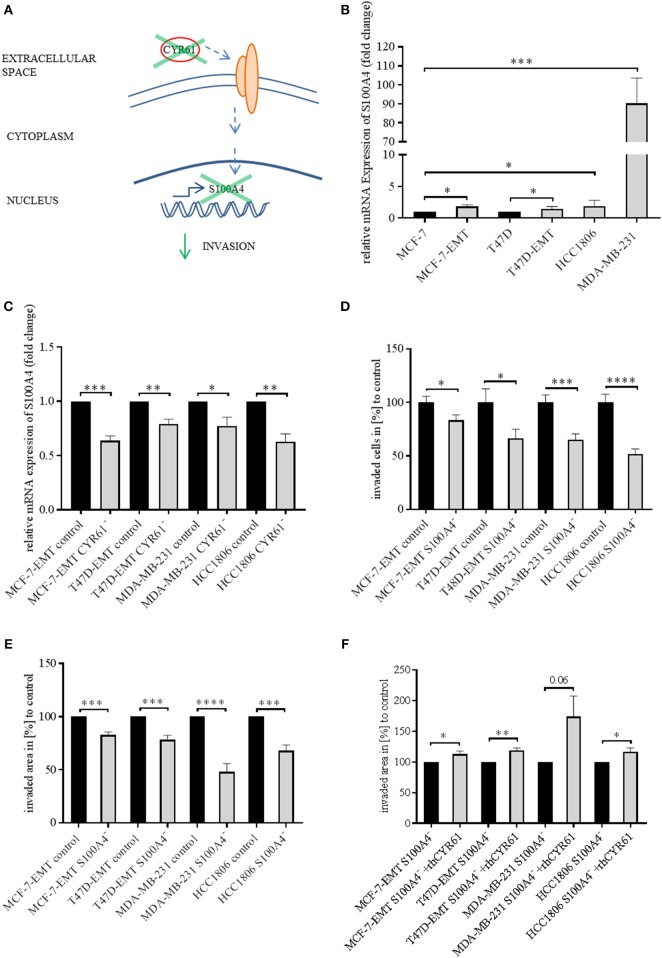
Suppression of CYR61 reduces S100A4 expression. **(A)** Scheme illustrating hypothesis of CYR61-dependent cell invasion regulation. **(B)** Relative S100A4 expression of invasive breast cancer cell lines compared to non-invasive controls. Data represent mean ± SEM. Using unpaired, two-tailed *t*-test analysis. MCF-7-EMT *n* = 5; T47D-EMT *n* = 5; MDA-MB-231 *n* = 4; HCC1806 *n* = 6; ^*^P < 0.05*;* ^***^P < 0.005 **(C)** Relative S100A4 expression of invasive breast cancer cell lines 96 h after transient CYR61 transfection compared to non-invasive controls. Data represent mean ± SEM. Using unpaired, two-tailed *t*-test analysis. MCF-7-EMT *n* = 4; T47D-EMT *n* = 3; MDA-MB-231 *n* = 4; HCC1806 *n* = 3; ^*^P < 0.05; ^**^P < 0.01; ^***^P < 0.005 **(D)** 2D Invasion analysis of co-cultured (MG-63, osteosarcoma cells) breast cancer cells transient transfected with S100A4 siRNA for 96 h. Percentage of cell invasion compared to controls was assessed by counting invaded cells under the filter in 4 random filter regions. Data represent mean ± SEM. MCF-7-EMT *n* = 36; T47D-EMT *n* = 36; MDA-MB-231 *n* = 24; HCC1806 *n* = 36; ^*^ *P* < 0.05; ^***^P < 0.005; ^****^ *P* < 0.0001 **(E)** 3D invasion analysis of breast cancer spheroids seeded after transient siRNA transfection. Spheroid area was assessed 48 h after adding Matrigel using polygonal selection and compared to spheroid area at time point 0 (adding of Matrigel). Area growth was compared to area growth of control spheroids. Data represent mean ± SEM. Using unpaired, two-tailed *t*-test analysis. MCF-7-EMT *n* = 6; T47D-EMT *n* = 6; MDA-MB-231 *n* = 12; HCC1806 *n* = 6; ^***^P < *0.005;* ^****^P < 0.0001 **(F)** 3D invasion analysis of breast cancer spheroids seeded after transient S100A4 siRNA transfection and treated with rhCYR61. Spheroid area was assessed 48 h after adding Matrigel and rhCYR61 using polygonal selection and compared to spheroid area at time point 0 (adding of Matrigel + rhCYR61). Area growth was compared to area growth of S100A4- spheroids. Data represent mean ± SEM. Using unpaired, two-tailed *t*-test analysis. MCF-7-EMT *n* = 4; T47D-EMT *n* = 4; MDA-MB-231 *n* = 5; HCC1806 *n* = 6; ^*^P < 0.05*;* ^**^P < 0.001.

Identically to CYR61, S100A4 is upregulated during EMT programs in breast cancer and correlates with bone metastasis (23, 25). Blocking extracellular signaling of S100A4 reduced invasiveness of breast cancer cells in a 2D transwell invasion assay (23). Both CYR61 and S100A4 alter breast cancer invasiveness but the underlying molecular mechanisms remain elusive. Chen et al. suggested that CTGF regulates S100A4 through regulation of extracellular regulated kinases ERK1 and ERK2 (26). CYR61 and CTGF both bind to integrinαV (12, 26, 27). We wanted to elucidate, whether suppression of CYR61 decreases S100A4 expression (Figure 2A). S100A4 was upregulated in mesenchymal transformed and TNBC cells (Figure 2B; MCF-7-EMT: 1.84 ± 0.27 SEM relative expression compared to MCF-7;*n* = 5; *P* = 0.014; T47D-EMT: 1.47 ± 0.16 SEM relative expression compared to T47D;*n* = 5; *P* = 0.0185; HCC1806: 1.89 ± 0.38 relative expression compared to MCF-7; *n* = 6; *P* = 0.0403; MDA-MB-231: 90.31 ± 13.3 SEM relative expression compared to MCF-7; *n* = 4; *P* = 0.0005). To elucidate the impact of CYR61 expression on S100A4, relative S100A4 expression was assessed after transient CYR61 suppression. Decreased CYR61 expression resulted in decreased S100A4 expression (Figure 2C; MCF-7-EMT CYR61^−^:0.64 ± 0.05 SEM relative S100A4 expression compared to MCF-7-EMT control; *n* = 4; *P* = 0.0002; T47D-EMT CYR61^−^: 0.79 ± 0.04 SEM relative S100A4 expression compared to T47D-EMT control; *n* = 3; *P* = 0.0078; MDA-MB-231 CYR61^−^:0.78 ± 0.08 SEM relative S100A4 expression compared to MDA-MB-231 control; *n* = 4; *P* = 0.0297; HCC1806 CYR61-: 0.63 ± 0.07 SEM relative S100A4 expression compared to HCC1806 control; *n* = 3; *P* = 0.0066), while suppresses S100A4 had no impact on CYR61 expression (Figure 2, Figure S3D). We investigated whether decreased S100A4 suppresses cell invasion in a 2D transwell co-culture assay. Decreased S100A4 expression (verification Figure 2, Figures S1–S3) suppressed the invasiveness of mesenchymal transformed und TNBC cells (Figure 2D; MCF-7-EMT S100A4^−^: 83.81 ± 4.9 SEM invaded cell in % compared to MCF-7-EMT control; *n* = 36; *P* = 0.0321; T47D-EMT S100A4^−^:66.29 ± 8.52 SEM invaded cells in % to T47D-EMT control; *n* = 36; *P* = 0.0303; MDA-MB-231 S100A4^−^:65.02 ± 5.58 SEM invaded cells in % to MDA-MB-231 control; *n* = 24; *P* = 0.0003; HCC1806 S100A4^−^: 51.84 ± 4.62 invaded cells in % to HCC1806 control; *n* = 36; *P* <0.0001). Furthermore, decreased S100A4 expression reduces 3D spheroid invasion area of mesenchymal transformed and TNBC cells (Figure 2E; MCF-7-EMT S100A4^−^: 82.77 ± 2.82 SEM invaded area in % compared to MCF-7-EMT control; *n* = 6; *P* = 0.0001; T47D-EMT S100A4^−^: 78.24 ± 4.17 SEM invaded area in % to T47D-EMT control; *n* = 6; *P* = 0.0004; MDA-MB-231 S100A4^−^: 47.93 ± 7.95 SEM invaded area in % to MDA-MB-231 control; *n* = 12; *P* < 0.0001; HCC1806 S100A4^−^: 67.97 ± 5.46 invaded area in % to HCC1806 control; *n* = 6; *P* = 0.0002), while proliferation was not altered (Figure 2, Figure S3E). To assess whether extracellular CYR61 can counteract the S100A4 suppressive effect on 3D spheroid invaded area, spheroids with suppressed S100A4 were treated with rhCYR61. Decreased S100A4 expression and additional increased extracellular CYR61 expression lead to an increased spheroid invaded area (Figure 2F; MCF-7-EMT S100A4^−^+rhCYR61: 112.8 ± 4.97 SEM invaded area in % compared to MCF-7-EMT S100A4^−^; *n* = 4; *P* = 0.0415; T47D-EMT S100A4^−^+rhCYR61: 118.9 ± 4.36 SEM invaded area in % compared to T47D-EMT S100A4^−^; *n* = 6; *P* = 0.0015; MDA-MB-231 S100A4^−^+rhCYR61: 174.2 ± 33.83 invaded area in % compared to MDA-MB-231 S100A4^−^; *n* = 5; *P* = 0.0596; HCC1806 S100A4^−^+rhCYR61: 116.3 ± 6.85 invaded area in % compared to HCC1806 S100A4^−^; *n* = 6; *P* = 0.0383). These data indicate a close correlation between CYR61 and S100A4 expression and the invasiveness of mesenchymal transformed and TNBC cells *in vitro*.

### ERK1/2 Activity Is Transducer of CYR61 Mediated S100A4 Regulation

**Figure 3:**
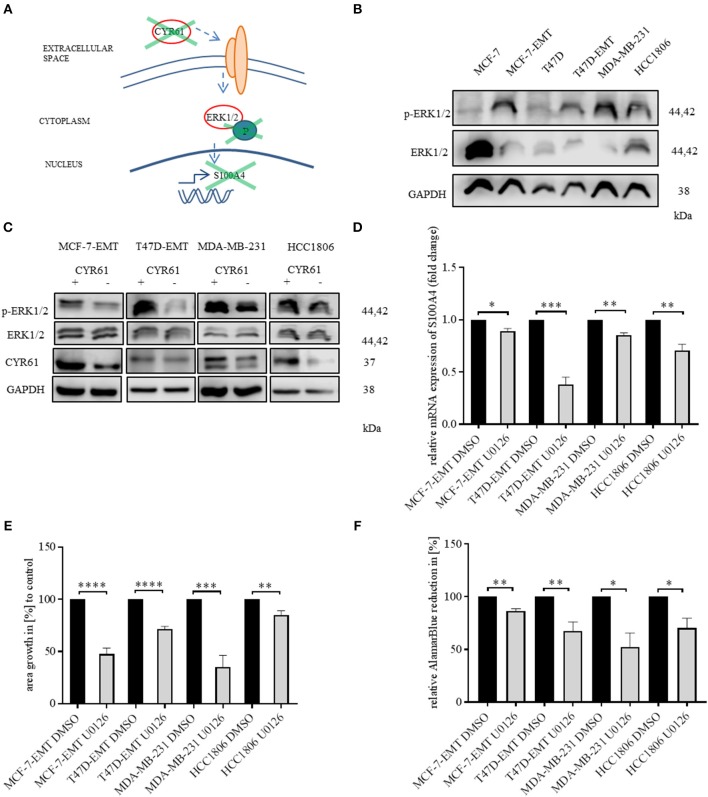
ERK1/2 activity is transducer of CYR61 mediated S100A4 regulation. **(A)** Scheme illustrating hypothesis of CYR61 regulating S100A4 in a p-ERK1/2 dependent manner. **(B)** ERK1/2 and p-Erk1/2 (Thr202/Tyr204) expression in different breast cancer cell lines detected by western blotting. **(C)** ERK1/2 and p-Erk1/2 (Thr202/Tyr204) and CYR61 expression in different breast cancer cell lines after transient CYR61 transfection detected by western blotting. **(D)** Relative S100A4 expression of invasive breast cancer cell lines treated with 10 μM U0126 compared to DMSO controls. Data represent mean ± SEM. Using unpaired, two-tailed *t*-test analysis. *n* = 3; ^*^P < 0.05*;* ^**^P < 0.01*;* ^***^P < 0.005 **(E)** 3D invasion analysis of breast cancer spheroids seeded after U0126 treatment. Spheroid area was assessed 48 h after adding Matrigel using polygonal selection and compared to spheroid area at time point 0 (adding of Matrigel + 10 μM U0126). Area growth was compared to area growth of control spheroids. Data represent mean ± SEM. Using unpaired, two-tailed *t*-test analysis. MCF-7-EMT *n* = 6; T47D-EMT *n* = 5; MDA-MB-231 *n* = 6; HCC1806 *n* = 5; ^**^P < 0.01*;* ^***^P < 0.005*;* ^****^P < 0.0001 **(F)** Analysis of relative AlamarBlue reduction as indicator for cell viability. Breast cancer cell spheroids were grown and AlamarBlue reduction was assessed 48 h after adding Matrigel and 10 μM U0126 at 4 h incubation. Relative AlamarBlue reduction was calculated compared to DMSO control spheroids. Data represent mean ± SEM. MCF-7-EMT *n* = 3; T47D-EMT *n* = 4; MDA-MB-231 *n* = 3; HCC1806 *n* = 3; ^*^P < 0.05*;* ^**^P < 0.01.

We found that decreased CYR61 resulted in a decreased S100A4 expression. Despite it remains elusive how CYR61 regulates S100A4 expression. To elucidate underlying intracellular mechanism we tested, whether decreased CYR61 expression reduces the phosphorylation of ERK1/2 thereby regulating S100A4 expression (Figure 3A). Mesenchymal transformed and TNBC cells shows a decreased ERK1/2 expression, while ERK1/2 phosphorylation was increased compared to non-invasive estrogen positive breast cancer cells (Figure 3B). Reducing CYR61 expression led to a decreased ERK1/2 phosphorylation (Figure 3C). MEK1 and MEK2 are upstream regulators of ERK1/2 activity (28) By using U0126 inhibitor, ERK phosphorylation can be diminished (29). Blocking ERK1/2 phosphorylation due to an MEK1 and MEK2 specific inhibitor U0126 (verification of U0126 induced blocking of ERK1/2 phosphorylation Figure 3, Figure S4A) resulted in a decreased S100A4 expression (Figure 3D; MCF-7-EMT U0126: 0.89 ± 0.02 SEM relative S100A4 expression compared to MCF-7-EMT DMSO; *n* = 3; *P* = 0.0114; T47D-EMT U0126: 0.38 ± 0.07 SEM relative S100A4 expression compared to T47D-EMT DMSO control; *n* = 3; *P* = 0.0009; MDA-MB-231 U0126: 0.85 ±0.02 SEM relative S100A4 expression compared to MDA-MB-231 DMSO; *n* = 3; *P* = 0.0026; HCC1806 U0126: 0.71 ± 0.06 SEM relative S100A4 expression compared to HCC1806 DMSO; *n* = 3; *P* = 0.0076). Furthermore, U0126 treatment reduced 3 D spheroid invaded area (Figure 3E; MCF-7-EMR U0126: 47.52 ± 5.77 SEM invaded area in % compared to MCF-7-EMT DMSO; *n* = 6; *P* < 0.0001; T47D-EMT U0126: 71.51 ± 2.61 SEM invaded area in % compared to T47D-EMT DMSO; *n* = 5; *P* < 0.0001; MDA-MB-231 U0126: 35.31 ± 10.91 SEM invaded area in % compared to MDA-MB-231 DMSO; *n* = 6; *P* = 0.0002; HCC1806 U0126: 85.01 ± 4.05 SEM invaded area in % compared to HCC1806 DMSO; *n* = 5; *P* = 0.006). Treatment with U0126 reduced proliferation in 3D spheroids (Figure 3F; MCF-7-EMT U0126: 86.57 ± 2.11 SEM relative AlamarBlue reduction in % compared to MCF-7-EMT DMSO; *n* = 3; *P* = 0.0031; T47D-EMT U0126: 67.53 ± 8.61 SEM relative AlamarBlue reduction compared to T47D-EMT DMSO; *n* = 4; *P* = 0.0093; MDA-MB-231 U0126:52.23 ± 13.32 SEM relative AlamarBlue reduction in % compared to MDA-MB-231 DMSO; *n* = 3; *P* = 0.023; HCC1806 U0126: 70.37 ± 9.29 SEM relative AlamarBlue reduction in % compared to HCC1806 DMSO; *n* = 3; *P* = 0.0332). Moreover, treating non-invasive estrogen positive breast cancer cell spheroids with rhCYR61 lead to increased ERK1/2 phosphorylation (Figure 3, Figure S4B). These results suggest that decreased ERK1/2 phosphorylation suppresses S100A4 expression. Moreover, ERK1/2 phosphorylation is reduced by decreased CYR61 expression.

### Suppression of YAP1 Reduces Invasiveness Through Altering CYR61-S100A4-pERK1/2 Signaling

**Figure 4:**
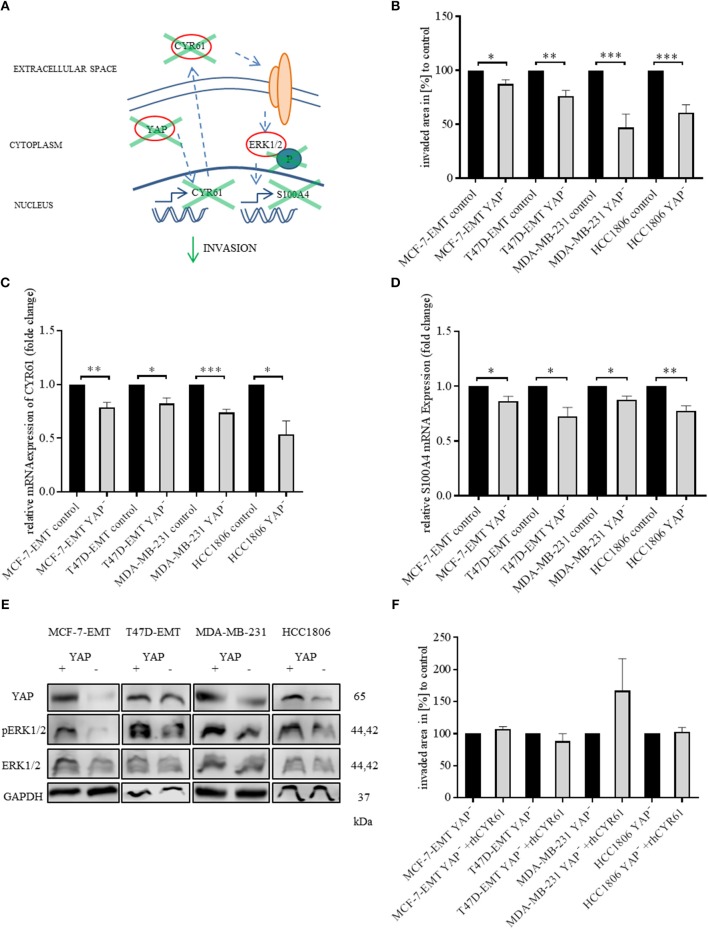
Suppression of YAP reduces invasiveness through blocking CYR61-S100A4-pERK1/2 signaling. **(A)** Scheme illustrating hypothesis of YAP regulating S100A4 in a CYR61 - p-ERK1/2 dependent manner. **(B)** 3D invasion analysis of breast cancer spheroids seeded after transient YAP siRNA transfection. Spheroid area was assessed 48 h after adding Matrigel using polygonal selection and compared to spheroid area at time point 0 (adding of Matrigel). Area growth was compared to area growth of control spheroids. Data represent mean ± SEM. Using unpaired, two-tailed *t*-test analysis. MCF-7-EMT *n* = 4; T47D-EMT *n* = 5; MDA-MB-231 *n* = 12; HCC1806 *n* = 8; ^*^P < 0.05*;* ^**^P < 0.01*;* ^***^P < 0.005 **(C)** Relative CYR61 expression of invasive breast cancer cell lines 48 h after transient YAP siRNA compared to controls. Data represent mean ± SEM. Using unpaired, two-tailed *t*-test analysis. *n* = 3;^*^P < 0.05*;* ^**^P < 0.01*;* ^***^P < 0.005 **(D)** Relative S100A4 expression of invasive breast cancer cell lines 48 h after transient YAP siRNA compared to controls. Data represent mean ± SEM. Using unpaired, two-tailed *t*-test analysis. *n* = 3; ^*^P < 0.05*;* ^**^P < 0.01; **(E)** ERK1/2, p-Erk1/2 (Thr202/Tyr204) and YAP expression in different breast cancer cell lines after transient YAP siRNA transfection detected by western blotting. **(F)** 3D invasion analysis of breast cancer spheroids seeded after transient YAP siRNA transfection and treated with rhCYR61. Spheroid area was assessed 48 h after adding Matrigel and rhCYR61 using polygonal selection and compared to spheroid area at time point 0 (adding of Matrigel + rhCYR61). Area growth was compared to area growth of YAP- spheroids. Data represent mean ± SEM. Using unpaired, two-tailed *t*-test analysis. MCF-7-EMT *n* = 6; T47D-EMT *n* = 6; MDA-MB-231 *n* = 4; HCC1806 *n* = 6.

Yes-associated protein (YAP) is a known upstream target of CYR61 in breast cancer (30). Validating that the observed results can be reproduced by altering YAP expression (Figure 4A), YAP was transiently decreased (verification Figure 4, Figure S5A). Decreased YAP expression reduced invaded area of 3 D spheroids (Figure 4B; MCF-7-EMT YAP^−^:87.48 ± 3.84 SEM invaded area in % compared to MCF-7-EMT control; *n* = 4; *P* = 0.0172; T47D-EMT YAP^−^: 76.23 ± 5.1 SEM invaded area in % compared to T47D-EMT control; *n* = 5; *P* = 0.0016; MDA-MB-231 YAP^−^: 47 ± 12.39 SEM invaded area in % compared to MDA-MB-231 control; *n* = 12; *P* = 0.0003; HCC1806 YAP^−^: 60.67 ± 7.38 SEM invaded area in % compared to HCC1806 control), while proliferation was not altered (Figure 4, Figure S5B). Decreased YAP expression reduces CYR61 expression (Figure 4C; MCF-7-EMT YAP^−^: 0.79 ± 0.05 SEM relative CYR61 expression compared to MCF-7-EMT control; *n* = 3; *P* = 0.01; T47D-EMT YAP^−^: 0.82 ± 0.05 SEM relative CYR61 expression compared to T47D-EMT control; *n* = 3; *P* = 0.0269; MDA-MB-231 YAP^−^: 0.74 ± 0.03 SEM relative CYR61 expression compared to MDA-MB-231 control; *n* = 3; *P* = 0.0008; HCC1806 YAP^−^: 0.54 ± 0.12 SEM relative CYR61 expression compared to HCC1806 control; *n* = 3; *P* = 0.0198) and S100A4 expression (Figure 4D; MCF-7-EMT YAP^−^: 0.86 ± 0.04 SEM relative S100A4 expression compared to MCF-7-EMT control; *n* = 3; *P* = 0.0362; T47D-EMT YAP^−^: 0.72 ± 0.08 SEM relative S100A4 expression compared to T47D-EMT control; *n* = 3; *P* = 0.0289; MDA-MB-231 YAP^−^: 0.88 ± 0.03 SEM relative S100A4 expression compared to MDA-MB-231 control; *n* = 3; *P* = 0.0179; HCC1806 YAP^−^: 0.78 ± 0.04 SEM relative S100A4 expression compared to HCC1806 control; *n* = 3; *P* = 0.0067). Furthermore, decreased YAP expression reduces ERK1/2 phosphorylation (Figure 4E). Transient decreased YAP expression in mesenchymal transformed and TNBC cells treated with rhCYR61 show no impact on spheroid invaded area (Figure 4F). Collectively, these data suggest that decreased YAP expression leads to a CYR61, pERK1/2 and S100A4 suppression. The effect of decreased YAP expression on spheroid invaded area can be restored be supplemented extracellular CYR61.

### CYR61 and S100A4 as Prognostic Markers for Invasive and Metastatic Breast Cancer

**Figure 5:**
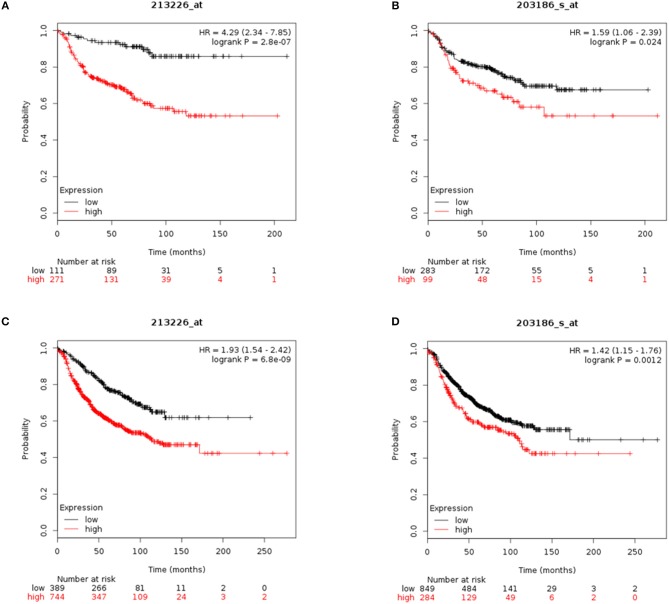
CYR61 and S100A4 as prognostic marker for breast cancer progression. **(A)** Probability of distant metastasis free survival (DMFS) in 382 breast cancer patients with lymph node positive status according to CYR61expression level. Kaplan–Meier plots were generated using Kaplan–Meier plotter (www.kmplot.com) data set 213226_at with a false-discovery rate (FDR) of 1%. Black line illustrates high CYR61 expression group and red line illustrates low CYR61 expression group. **(B)** Probability of DMFS in 382 breast cancer patients with lymph node positive status according to S100A4 expression level. Kaplan–Meier plots were generated using Kaplan–Meier plotter (www.kmplot.com) data set 203186_at with a false-discovery rate (FDR) over 50%. Black line illustrates high S100A4 expression group and red line illustrates low S100A4 expression group. **(C)** Probability of remission free survival (RFS) in 1133 breast cancer patients with lymph node positive status according to CYR61 expression level. Kaplan–Meier plots were generated using Kaplan–Meier plotter (www.kmplot.com) data set 213226_at with a false-discovery rate (FDR) of 1%. Black line illustrates high CYR61 expression group and red line illustrates low CYR61 expression group. **(D)** Probability of RFS in 1133 breast cancer patients with lymph node positive status according to CYR61 expression level. Kaplan–Meier plots were generated using Kaplan–Meier plotter (www.kmplot.com) data set 203186_at with a false-discovery rate (FDR) over 50 %. Black line illustrates high S100A4 expression group and red line illustrates low S100A4 expression group. HR, hazard ratio.

To assess the value of CYR61 and S100A4 as prognostic marker meta-analysis were conducted. Reduced CYR61 expression increases the probability of distant-metastasis free survival (DMFS) of breast cancer patients with a lymph node positive status (Figure 5A; dataset 213226_at; *n* = 382; FDR 1%; *P* = 2.8 e^−07^). Reduced S100A4 expression increases the probability of DMFS of breast cancer patients with a lymph node positive status but shows a higher FDR (Figure 5B; dataset 203186_s_at; *n* = 382; FDR > 50%; *P* = 0.024, cut-off values see Figure 5, Figure S6). Analyzing the effects of decreases CYR61 or S100A4 expression with regards to the relapse free survival (RFS) shows comparable results (Figures 5C,D; CYR61: dataset 213226_at; *n* = 1133; FDR 1%; *P* = 6.8 e-^09^; S100A4: dataset 203186_s_at; FDR > 50%; *P* = 0.0012, cut-off values see Figure 5, Figure S6). These data demonstrate that CYR61 could act as a prognostic marker in breast cancer.

### CYR61 and S100A4 as Therapeutic Target for Invasive and Metastatic Breast Cancer

**Figure 6:**
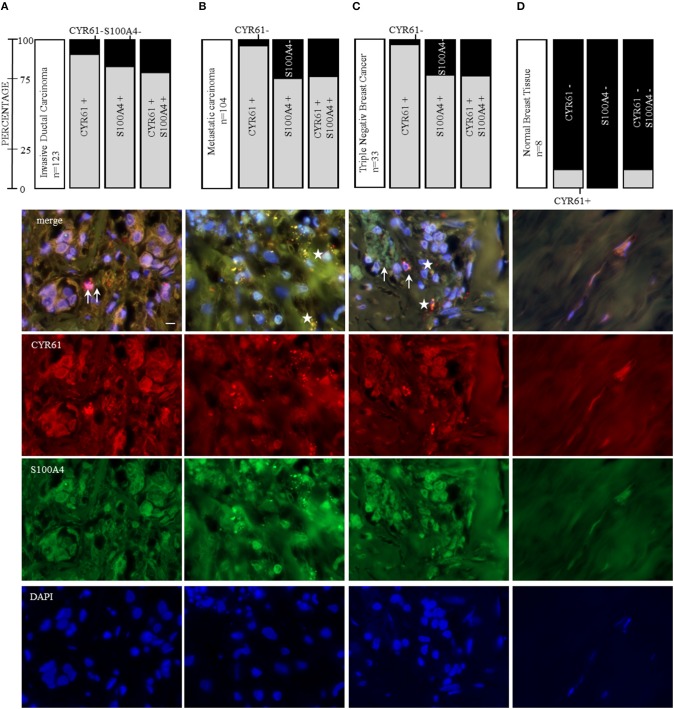
CYR61 and S100A4 as therapeutic target for invasive and metastatic breast cancer. Immunofluorescence staining of three tissue arrays (biomax) containing 104 invasive ductal carcinoma tissue sections with corresponding metastatic carcinomas, further 21 invasive ductal carcinoma tissue sections and 8 normal breast tissue sections. With 33 of the carcinoma tissue sections being negative for the estrogen receptor and progesterone receptor and do not overexpress the Her2neu receptor (triple negative breast cancer, TNBC). **(A)** CYR61 and/or S100A4 expression analysis in 123 invasive ductal carcinoma tissue sections and corresponding lymph node sections from 104 patients **(B)** and representative immunofluorescence staining analyzed with a 100x oil objective (Axio ZEISS). **(C)** Within the 123 invasive ductal carcinomas patient tissue sections 33 were stated as being TNBC. **(D)** Normal breast tissue sections (*n* = 8) were analyzed for their CYR61 and/ or S100A4 expression using immunofluorescence staining. Scale bar gauges 20 μm.

CYR61 and S100A4 are drivers for breast cancer cell invasion *in vitro*. Consequently, we examined the value of CYR61 and/or S100A4 as a potential therapeutic target for advanced breast cancer. Analyzing the expression in 239 paraffin-fixed patient breast tissue sections (104 invasive breast cancer sections with corresponding metastatic lymph node section and progesterone receptor-, estrogen receptor- and Her2neu expression, BR20837; 17 invasive ductal,1 medullary carcinoma and 6 normal breast tissue sections, BR248a; 2 invasive ductal carcinomas, 1 invasive lobular carcinoma and 2 normal breast tissue section, T087a). Analyzing if expression was detected (immunofluorescence signal for CYR61 and/or S100A4 1-5 spots +; 5-10 spots ++; >10 spots +++) or not (–), we find the following pattern: 90.2% of invasive ductal carcinomas were positive for CYR61 expression, 82% were positive for S100A4 expression and 78% showed both CYR61 and S100A4 expression (Figure 6A and Figure S7). Corresponding metastatic lymph node sections were in 96% positive for CYR61, in 75% positive for S100A4 and in 74% for both CYR61 and S100A4. TNBC tissue sections were in 97% positive for CYR61, in 75.8% positive for S100A4, and in 75.8% expressing both CYR61 and S100A4. Interestingly, CYR61 expression was only detected in 12.5% of normal breast tissue samples and S100A4 expression in none (Figure 6D, detailed list Figure S7). Visual expression of CYR61 and S100A4 in blood vessels (Figure 6D) could be found throughout all tissue sections. We find that the CYR61 and S100A4 expression appeared in very close localization to each other (Figure 6, white arrows) or even co-localized (Figure 6, white stars). These studies demonstrate that CYR61 and S100A4 could be valuable therapeutic targets and prognostic marker for invasive breast cancer and metastasis.

## Discussion

CYR61 is best recognized as regulator of inflammation and wound healing (31, 32). Several studies indicate that CYR61 can facilitate invasion and is crucial for EMT programs regarding cancer progression (12, 15, 16, 23). The question remains how CYR61 facilitates invasion in breast cancer and which role it possesses regarding EMT complexity (4). Since CYR61 has known oncogenic functions in serval tumor entities (12, 13), including breast cancer (15, 16), the question appeared if CYR61 might be a valuable therapeutic target in aggressive breast cancer and if it could be a prognostic marker for these indications. We report that a higher CYR61 expression correlates with a poor prognosis of breast cancer patients. Moreover, we found that reducing the CYR61 expression leads to a decreased invasion in 2D and 3D invasion analysis setups, showing comparable results. Suggesting that reduced invasion upon CYR61 suppression is due to reduces ERK1/2 phosphorylation and S100A4 expression. CYR61 might be a valuable therapeutic target and prognostic marker for invasive and metastatic breast cancer.

Triple negative breast cancers (TNBC) account for 15–20% of all breast cancer incidents and there is no specific targeted therapy available (33). There is a need for identifying new targets for future therapy options. Consistent with previous published results we could demonstrate that CYR61 expression is increased in TNBC cell line MDA-MB-231 (34) and further more in the TNBC cell line HCC1806. The contribution of EMT-induced expression changes to the invasion and metastatic cascade regarding cancer progression is highly debated and needs to be interpreted cell and tissue specific (4, 8, 35). Mesenchymal transformed breast cancer cells show an increased expression of CYR61 and S100A4 (23), which we could reproduce in our setting. It was shown that S100A4 facilitates breast cancer invasion (36). Gründker et al. demonstrated that suppressing extracellular signaling of CYR61 and S100A4 decreased the ability of breast cancer cell invasion in a co-cultural setting mimicking bone metastasis (23). It was not tested how the intracellular signaling is affected when CYR61 or S100A4 expression is reduced. We report here that transient gene silencing of either CYR61 or S100A4 can reduce invasiveness in mesenchymal transformed and TNBC cells. To further assess the impact of CYR61 on breast cancer cell invasion we increased extracellular CYR61 expression in non-invasive breast cancer cells and could show that this led to an increased invasive behavior. These findings indicate that CYR61 could be a regulator of breast cancer cells invasion. We showed that reversing EMT-induced upregulation of CYR61 and S100A4 leads to reduced invasive behavior in breast cancer cells in different invasion setups. This could indicate a role of EMT within this process. Further research is necessary to assess, if modulating CYR61 regulates EMT-TFs and thereby facilitates cellular plasticity. It has been suggested that targeting EMT-TFs could help to overcome chemo resistance and recent findings suggest an involvement of CYR61 in resistance to certain therapies in different tumor entities (5, 37, 38).

Despite, it was unclear how CYR61 regulates invasiveness of breast cancer cells. We suggest that CYR61 regulates S100A4 expression in mesenchymal transformed and TNBC cells through regulating ERK1/2 phosphorylation. Reducing S100A4 expression leads to decreased 3 D spheroid invasion and invasiveness of breast cancer cells in co-culture with osteosarcoma cells. Adding extracellular CYR61 to breast cancer spheroids with transient decreased S100A4 expression could restore the effect und led to a slightly increased invaded area. Hou et al. suggested that regulating CYR61 in osteosarcoma cells targets the MEK-ERK pathway (12). ERK1/2 signaling is gaining higher interest since the unique ERK1/2 position within cellular signaling. Targeting ERK1/2 could be valuable for therapy-resistant cancer to known clinically used BRAF and MEK inhibitors (39). We could show that inhibition of ERK1/2 phosphorylation led to decreased 3D spheroid invasion and reduced spheroid proliferation. Inhibition of ERK1/2 phosphorylation led to decreased S100A4 expression. But S100A4 decreased expression by itself had no impact on spheroid proliferation, neither had CYR61 or YAP transient suppression.

YAP is regulated negatively through the Hippo-Pathway, which regulates key events of organ size, development and angiogenesis (40–42). Regarding breast cancer YAP is reported to have dual function as oncogene and tumor suppressor (43). Higher YAP expression correlates with increased EMT marker expression (44). We suggest that reduced YAP expression leads to decreased 3D spheroid invasion by suppression of CYR61, p-ERK1/2 and S100A4. The effect of reduced YAP expression on 3D invasion could be restored by extracellular CYR61 addition.

CYR61 or S100A4 are suggested to be valuable prognostic markers regarding several tumor entities (45–48). Egeland et al. suggested the use of S100A4 as a prognostic marker for early-stage breast cancer (49). We examined whether CYR61 and S100A4 could be valuable prognostic marker for invasive and metastatic breast cancer. CYR61 and S100A4 are highly expressed in invasive-ductal carcinomas, including TNBC, and both are expressed in metastatic lymph node sections. Of all analyzed tissue sections 82.2% expressing CYR61 did express S100A4, respectively, which lead to the conclusion, that CYR61 together with S100A4 would be valuable prognostic marker for breast cancer and breast cancer metastasis. Moreover, we found that expression of CYR61 and S100A4 is closely located (Figure 6, indicated by arrow) or even co-localized (Figure 6, indicated by star). Considering that CYR61 regulates cancer invasion and the findings, that it may be a valuable prognostic marker in different cancer entities (45, 46, 50, 51), It was suggested before, that CYR61 regulates E-cadherin, N-cadherin and Twist in osteosarcoma cells (12). Further investigations should clarify if CYR61 suppression regulates EMT-TFs in breast cancer and facilitates invasion by altering ECM degradation and adhesion. Secretome analysis of co-cultured cancer cells could identify secreted proteins, like matricellular proteins, that are drivers for invasion and promote metastasis.

Our findings suggest that CYR61 plays a major role in breast cancer invasion. This impact is facilitated through the regulation of ERK phosphorylation and S100A4 expression. Moreover, targeting YAP, a CYR61 upstream regulator, regulates CYR61, ERK phosphorylation and S100A4. We could identify a close correlation between CYR61 and S100A4 expression and breast cancer invasion and metastasis in breast cancer patients. CYR61 together with S100A4 might be utilized as therapeutic target and prognostic marker for invasive breast cancer and metastasis.

## Data Availability Statement

The datasets used and/or analyzed during the current study are available from the corresponding author on reasonable request.

## Author Contributions

Conception and design of the reported work was done by JH and CG. JH, GB, and CG did the development of methodology used. JH and SH performed invasion assays. JH and LG contributed to protein expression analysis. JH contributed to immune histochemical staining, gene expression analysis, proliferation analysis, *in silico* and network analysis. Analysis and interpretation of data was performed by JH, SH, LG, GB, GE, and CG. All authors read and approved the final manuscript.

### Conflict of Interest

The authors declare that the research was conducted in the absence of any commercial or financial relationships that could be construed as a potential conflict of interest.

## Supplementary Material

Figure S1 CYR61 expression correlates with breast cancer cell invasiveness. **(A)** Relative transforming growth factor beta induced (TGFBI) expression of mesenchymal transformed breast cancer cell lines compared to non-invasive controls was assessed using real-time quantitative PCR. Data represent mean ± SEM. Using unpaired, two-tailed *t*-test analysis. MCF-7-EMT *n* = 3; T47D-EMT *n* = 6; ^***^*P* < 0.0005*;* ^****^*P* < 0.0001 **(B)** Relative E-cadherin expression of mesenchymal transformed breast cancer cell lines compared to non-invasive controls was assessed using real-time quantitative PCR. Data represent mean ± SEM. Using unpaired, two-tailed *t*-test analysis. MCF-7-EMT *n* = 4; T47D-EMT *n* = 3; ^*^*P* < 0.05*;* ^****^*P* < 0.0001 **(C)** Relative Vimentin expression of mesenchymal transformed breast cancer cell lines compared to non-invasive controls was assessed using real-time quantitative PCR. Data represent mean ± SEM. Using unpaired, two-tailed *t*-test analysis. MCF-7-EMT *n* = 5; T47D-EMT *n* = 3; ^*^*P* < 0.05 **(D)** Relative Zeb1 expression of mesenchymal transformed breast cancer cell lines compared to non-invasive controls was assessed using real-time quantitative PCR. Data represent mean ± SEM. Using unpaired, two-tailed *t*-test analysis. MCF-7-EMT *n* = 4; T47D-EMT *n* = 3; ^*^*P* < 0.05 **(E)** Relative SNAI1 expression of mesenchymal transformed breast cancer cell lines compared to non-invasive controls was assessed using real-time quantitative PCR. Data represent mean ± SEM. Using unpaired, two-tailed *t*-test analysis. MCF-7-EMT *n* = 4; T47D-EMT *n* = 5; ^***^*P* < 0.0005 **(F)** Relative SNAI2 expression of mesenchymal transformed breast cancer cell lines compared to non-invasive controls was assessed using real-time quantitative PCR. Data represent mean ± SEM. Using unpaired, two-tailed *t*-test analysis. MCF-7-EMT *n* = 3; T47D-EMT *n* = 4; ^*^*P* < 0.05.

Figure S2 CYR61 expression correlates with breast cancer cell invasiveness. **(A)** Relative CYR61 expression 96 h after transient CYR61 siRNA transfection compared to control was assessed using real-time quantitative PCR. Data represent mean ± SEM. Using unpaired, two-tailed *t*-test analysis. MCF-7-EMT *n* = 8; T47D-EMT *n* = 7; MDA-MB-231 *n* = 3; HCC1806 *n* = 4; ^**^ *P* < 0.01; ^***^*P* < 0.0001 **(B)** Analysis of relative AlamarBlue reduction as indicator for cell viability. Transient transfected breast cancer cell spheroids were grown and AlamarBlue reduction was assessed 48 hours after adding Matrigel at 4 h incubation. Relative AlamarBlue reduction was calculated compared to control spheroids. Data represent mean ± SEM. *n* = 3 **(C)** Analysis of relative AlamarBlue reduction as indicator for cell viability. Breast cancer cell spheroids were grown and AlamarBlue reduction was assessed 48 h after adding Matrigel and 1μg/ml rhCYR61 at 4 h incubation. Relative AlamarBlue reduction was calculated compared to control spheroids. Data represent mean ± SEM. *n* = 3.

Figure S3 Suppression of CYR61 reduces S100A4 expression. **(A)** Immunoblot analysis of S100A4 mRNA expression levels in different breast cancer cell lines 96 h after S100A4 siRNA transfection was detected using western blotting. Date represent mean ± SEM. Using unpaired, two-tailed *t*-test analysis. MCF-7-EMT *n* = 4; T47D-EMT *n* = 4; MDA-MB-231 *n* = 3; HCC1806 *n* = 3; ^*^*P* < 0.05*;* ^**^*P* < 0.01*;* ^***^*P* < 0.005 **(B)** Representative experiments of S100A4 protein expression quantification corresponding to **(A)**. **(C)** S100A4 mRNA expression analysis 96 h after siRNA transfection using quantitative PCR. Date represent mean ± SEM. Using unpaired, two-tailed *t*-test analysis. MCF-7-EMT *n* = 4; T47D-EMT *n* = 4; MDA-MB-231 *n* = 3; HCC1806 *n* = 3; ^***^*P* < 0.005; ^****^*P* < 0.0001 **(D)** CYR61 mRNA expression analysis 96 h after S100A4 siRNA transfection using quantitative PCR. Date represent mean ± SEM. MCF-7-EMT *n* = 5; T47D-EMT *n* = 6; MDA-MB-231 *n* = 3; HCC1806 *n* = 3 **(E)** Analysis of relative AlamarBlue reduction as indicator for cell viability. Breast cancer cell spheroids transient transfected with S100A4 siRNA were grown and AlamarBlue reduction was assessed 48 h after adding Matrigel at 4 h incubation. Relative AlamarBlue reduction was calculated compared to control spheroids. Data represent mean ± SEM. *n* = 3.

Figure S4 ERK1/2 activity is transducer of CYR61 mediated S100A4 regulation. **(A)** ERK1/2 and p-Erk1/2 (Thr202/Tyr204) expression in different breast cancer cell lines with or without 10μM U0126 treatment detected by western blotting. **(B)** ERK1/2 and p-Erk1/2 (Thr202/Tyr204) expression in non-invasive breast cancer cell lines with or without 1μg/ml rhCYR61 treatment detected by western blotting.

Figure S5 Suppression of YAP reduces invasiveness through blocking CYR61-S100A4-pERK1/2 signaling. **(A)** Relative YAP expression 96 h after transient YAP siRNA transfection compared to control was assessed using real-time quantitative PCR. Data represent mean ± SEM. Using unpaired, two-tailed *t*-test analysis. MCF-7-EMT *n* = 5; T47D-EMT *n* = 3; MDA-MB-231 *n* = 3; HCC1806 *n* = 3; ^*^*P* < 0.05*;* ^**^*P* < 0.01*;* ^****^*P* < 0.001 **(B)** Analysis of relative AlamarBlue reduction as indicator for cell viability. Breast cancer cell spheroids were grown and AlamarBlue reduction was assessed 48 hours after adding Matrigel at 4 h incubation. Relative AlamarBlue reduction was calculated compared to control spheroids. Data represent mean ± SEM. *n* = 3.

Figure S6 CYR61 and S100A4 as prognostic marker for breast cancer progression. Cut-off values were downloaded from kmplot.com after target (dataset 213226_at = CYR61; dataset 203186_s_at = S100A4) specific analysis. RFS, relapse free survival; DMFS, distant metastasis free survival.

Figure S7 CYR61 and S100A4 are highly expressed in invasive and metastatic B cancer patient tissue samples. Expression analysis of CYR61 and S100A4 via fluorescence staining using biomax tissue arrays (BR 20837,BR 248a, T087a) with paraffin-embedded patient samples. Table shows Arraytyp of analyzed samples, patients age, sex, the organic tissue site, pathology diagnosis, classification of M tumors (TNM), grading, stage, typ, tisse ID and for most analyzed tiised the xpression of estrogen(ER), progesteron (PR) and Herceptinreceptor2 (Her2). Expression of CYR61 and S100A4 was assessed as (−) not expressed, (+) low expression, (++) medium expression, (+++) high expression.
