# Supplementary figures and images for: Inhibition of CYR61-S100A4 Axis Limits Breast Cancer Invasion
Source: Front Oncol. 2019 Oct 23;9:1074. doi: 10.3389/fonc.2019.01074 (PMC6819319; doi:10.3389/fonc.2019.01074)

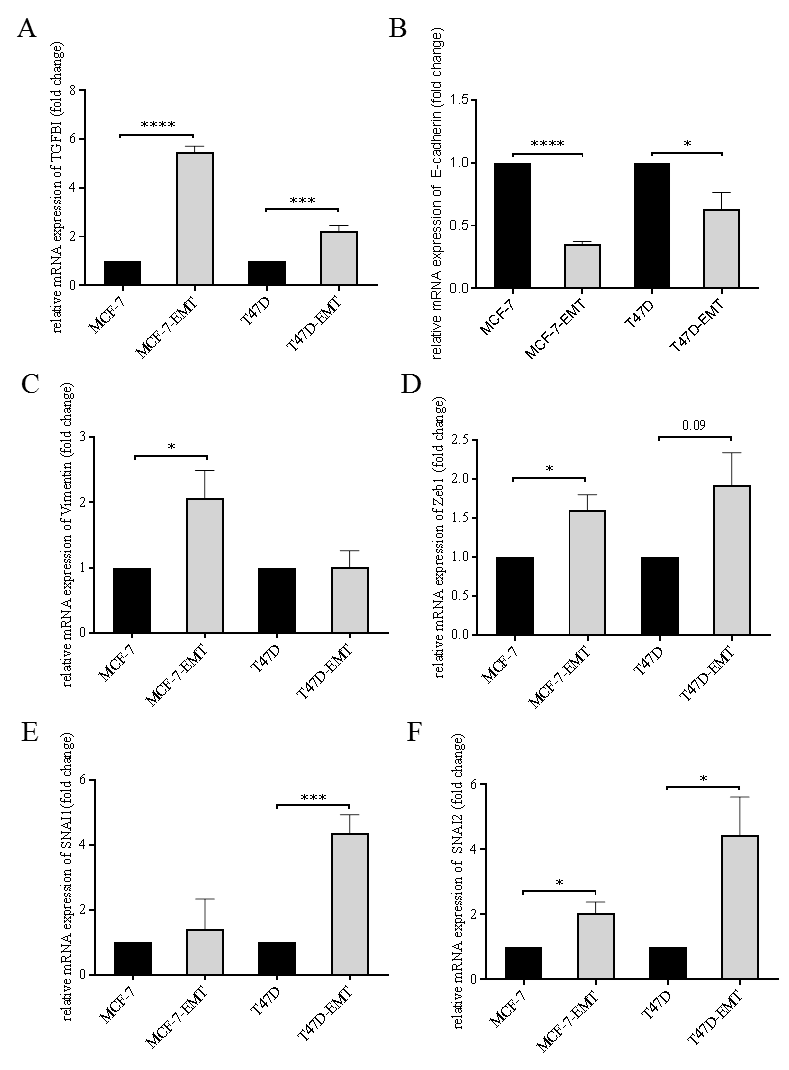

Supplement: Figure S1 — CYR61 expression correlates with breast cancer cell invasiveness. (A) Relative transforming growth factor beta induced (TGFBI) expression of mesenchymal transformed breast cancer cell lines compared to non-invasive controls was assessed using real-time quantitative PCR. Data represent mean ± SEM. Using unpaired, two-tailed t-test analysis. MCF-7-EMT n = 3; T47D-EMT n = 6; ***P < 0.0005; ****P < 0.0001 (B) Relative E-cadherin expression of mesenchymal transformed breast cancer cell lines compared to non-invasive controls was assessed using real-time quantitative PCR. Data represent mean ± SEM. Using unpaired, two-tailed t-test analysis. MCF-7-EMT n = 4; T47D-EMT n = 3; *P < 0.05; ****P < 0.0001 (C) Relative Vimentin expression of mesenchymal transformed breast cancer cell lines compared to non-invasive controls was assessed using real-time quantitative PCR. Data represent mean ± SEM. Using unpaired, two-tailed t-test analysis. MCF-7-EMT n = 5; T47D-EMT n = 3; *P < 0.05 (D) Relative Zeb1 expression of mesenchymal transformed breast cancer cell lines compared to non-invasive controls was assessed using real-time quantitative PCR. Data represent mean ± SEM. Using unpaired, two-tailed t-test analysis. MCF-7-EMT n = 4; T47D-EMT n = 3; *P < 0.05 (E) Relative SNAI1 expression of mesenchymal transformed breast cancer cell lines compared to non-invasive controls was assessed using real-time quantitative PCR. Data represent mean ± SEM. Using unpaired, two-tailed t-test analysis. MCF-7-EMT n = 4; T47D-EMT n = 5; ***P < 0.0005 (F) Relative SNAI2 expression of mesenchymal transformed breast cancer cell lines compared to non-invasive controls was assessed using real-time quantitative PCR. Data represent mean ± SEM. Using unpaired, two-tailed t-test analysis. MCF-7-EMT n = 3; T47D-EMT n = 4; *P < 0.05. [file Presentation_1.zip › S1.tif]

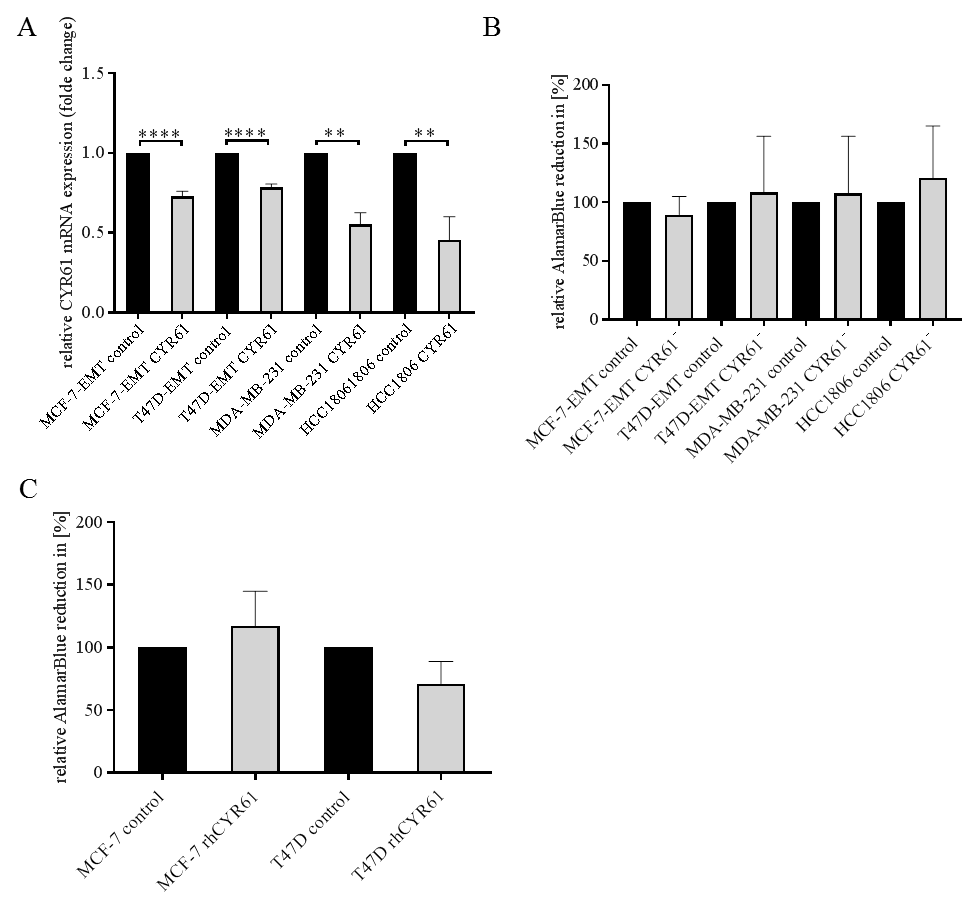

Supplement: Figure S1 — CYR61 expression correlates with breast cancer cell invasiveness. (A) Relative transforming growth factor beta induced (TGFBI) expression of mesenchymal transformed breast cancer cell lines compared to non-invasive controls was assessed using real-time quantitative PCR. Data represent mean ± SEM. Using unpaired, two-tailed t-test analysis. MCF-7-EMT n = 3; T47D-EMT n = 6; ***P < 0.0005; ****P < 0.0001 (B) Relative E-cadherin expression of mesenchymal transformed breast cancer cell lines compared to non-invasive controls was assessed using real-time quantitative PCR. Data represent mean ± SEM. Using unpaired, two-tailed t-test analysis. MCF-7-EMT n = 4; T47D-EMT n = 3; *P < 0.05; ****P < 0.0001 (C) Relative Vimentin expression of mesenchymal transformed breast cancer cell lines compared to non-invasive controls was assessed using real-time quantitative PCR. Data represent mean ± SEM. Using unpaired, two-tailed t-test analysis. MCF-7-EMT n = 5; T47D-EMT n = 3; *P < 0.05 (D) Relative Zeb1 expression of mesenchymal transformed breast cancer cell lines compared to non-invasive controls was assessed using real-time quantitative PCR. Data represent mean ± SEM. Using unpaired, two-tailed t-test analysis. MCF-7-EMT n = 4; T47D-EMT n = 3; *P < 0.05 (E) Relative SNAI1 expression of mesenchymal transformed breast cancer cell lines compared to non-invasive controls was assessed using real-time quantitative PCR. Data represent mean ± SEM. Using unpaired, two-tailed t-test analysis. MCF-7-EMT n = 4; T47D-EMT n = 5; ***P < 0.0005 (F) Relative SNAI2 expression of mesenchymal transformed breast cancer cell lines compared to non-invasive controls was assessed using real-time quantitative PCR. Data represent mean ± SEM. Using unpaired, two-tailed t-test analysis. MCF-7-EMT n = 3; T47D-EMT n = 4; *P < 0.05. [file Presentation_1.zip › S2.tif]

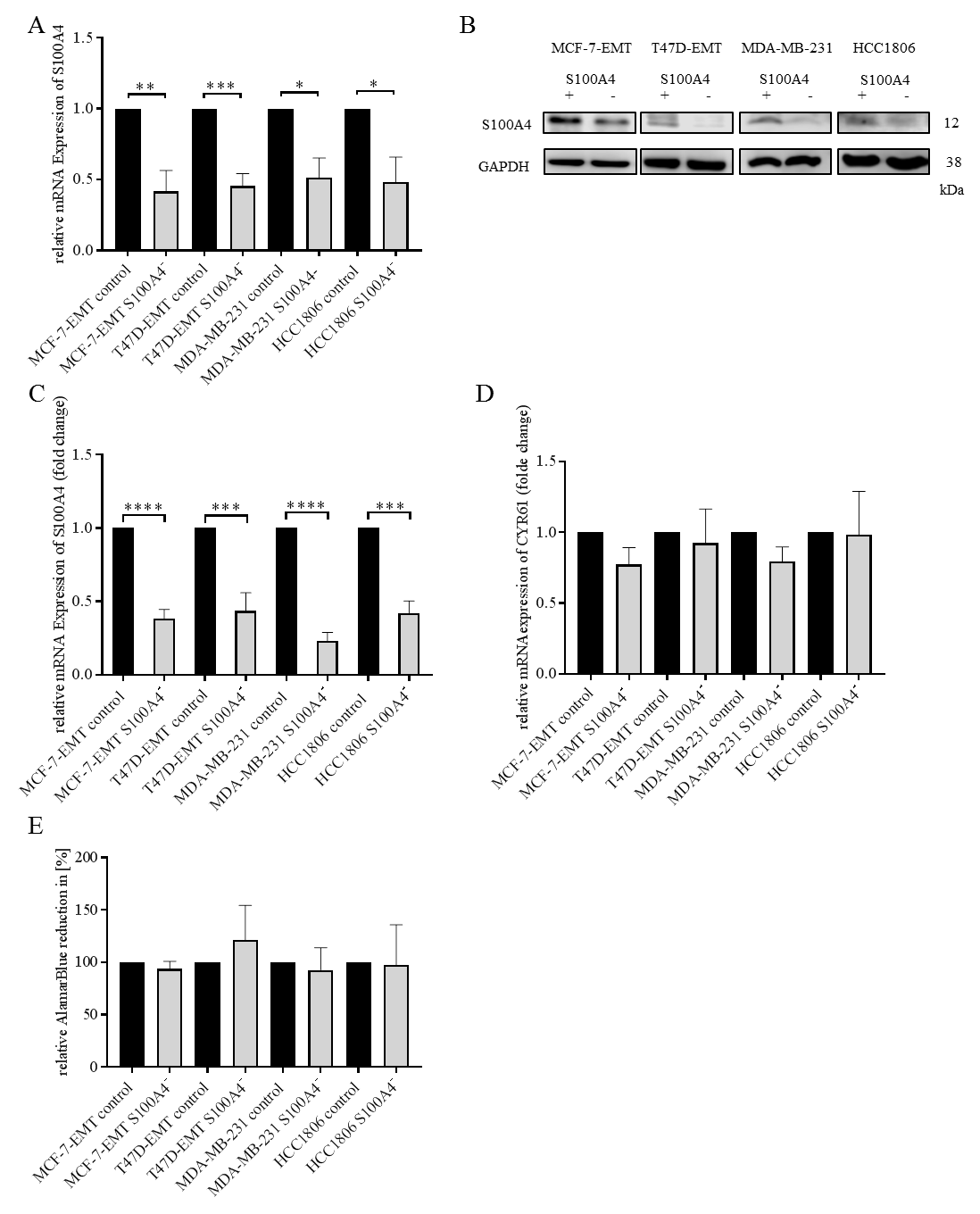

Supplement: Figure S1 — CYR61 expression correlates with breast cancer cell invasiveness. (A) Relative transforming growth factor beta induced (TGFBI) expression of mesenchymal transformed breast cancer cell lines compared to non-invasive controls was assessed using real-time quantitative PCR. Data represent mean ± SEM. Using unpaired, two-tailed t-test analysis. MCF-7-EMT n = 3; T47D-EMT n = 6; ***P < 0.0005; ****P < 0.0001 (B) Relative E-cadherin expression of mesenchymal transformed breast cancer cell lines compared to non-invasive controls was assessed using real-time quantitative PCR. Data represent mean ± SEM. Using unpaired, two-tailed t-test analysis. MCF-7-EMT n = 4; T47D-EMT n = 3; *P < 0.05; ****P < 0.0001 (C) Relative Vimentin expression of mesenchymal transformed breast cancer cell lines compared to non-invasive controls was assessed using real-time quantitative PCR. Data represent mean ± SEM. Using unpaired, two-tailed t-test analysis. MCF-7-EMT n = 5; T47D-EMT n = 3; *P < 0.05 (D) Relative Zeb1 expression of mesenchymal transformed breast cancer cell lines compared to non-invasive controls was assessed using real-time quantitative PCR. Data represent mean ± SEM. Using unpaired, two-tailed t-test analysis. MCF-7-EMT n = 4; T47D-EMT n = 3; *P < 0.05 (E) Relative SNAI1 expression of mesenchymal transformed breast cancer cell lines compared to non-invasive controls was assessed using real-time quantitative PCR. Data represent mean ± SEM. Using unpaired, two-tailed t-test analysis. MCF-7-EMT n = 4; T47D-EMT n = 5; ***P < 0.0005 (F) Relative SNAI2 expression of mesenchymal transformed breast cancer cell lines compared to non-invasive controls was assessed using real-time quantitative PCR. Data represent mean ± SEM. Using unpaired, two-tailed t-test analysis. MCF-7-EMT n = 3; T47D-EMT n = 4; *P < 0.05. [file Presentation_1.zip › S3.tif]

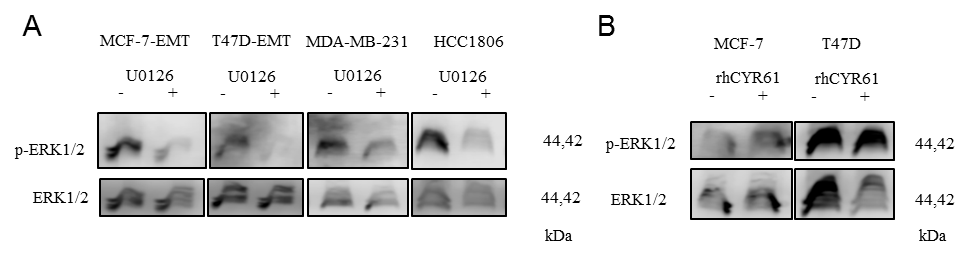

Supplement: Figure S1 — CYR61 expression correlates with breast cancer cell invasiveness. (A) Relative transforming growth factor beta induced (TGFBI) expression of mesenchymal transformed breast cancer cell lines compared to non-invasive controls was assessed using real-time quantitative PCR. Data represent mean ± SEM. Using unpaired, two-tailed t-test analysis. MCF-7-EMT n = 3; T47D-EMT n = 6; ***P < 0.0005; ****P < 0.0001 (B) Relative E-cadherin expression of mesenchymal transformed breast cancer cell lines compared to non-invasive controls was assessed using real-time quantitative PCR. Data represent mean ± SEM. Using unpaired, two-tailed t-test analysis. MCF-7-EMT n = 4; T47D-EMT n = 3; *P < 0.05; ****P < 0.0001 (C) Relative Vimentin expression of mesenchymal transformed breast cancer cell lines compared to non-invasive controls was assessed using real-time quantitative PCR. Data represent mean ± SEM. Using unpaired, two-tailed t-test analysis. MCF-7-EMT n = 5; T47D-EMT n = 3; *P < 0.05 (D) Relative Zeb1 expression of mesenchymal transformed breast cancer cell lines compared to non-invasive controls was assessed using real-time quantitative PCR. Data represent mean ± SEM. Using unpaired, two-tailed t-test analysis. MCF-7-EMT n = 4; T47D-EMT n = 3; *P < 0.05 (E) Relative SNAI1 expression of mesenchymal transformed breast cancer cell lines compared to non-invasive controls was assessed using real-time quantitative PCR. Data represent mean ± SEM. Using unpaired, two-tailed t-test analysis. MCF-7-EMT n = 4; T47D-EMT n = 5; ***P < 0.0005 (F) Relative SNAI2 expression of mesenchymal transformed breast cancer cell lines compared to non-invasive controls was assessed using real-time quantitative PCR. Data represent mean ± SEM. Using unpaired, two-tailed t-test analysis. MCF-7-EMT n = 3; T47D-EMT n = 4; *P < 0.05. [file Presentation_1.zip › S4.tif]

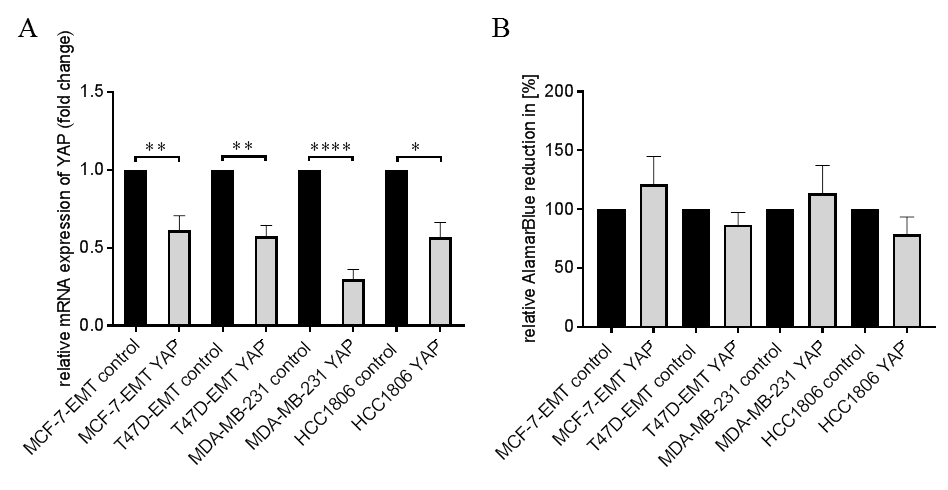

Supplement: Figure S1 — CYR61 expression correlates with breast cancer cell invasiveness. (A) Relative transforming growth factor beta induced (TGFBI) expression of mesenchymal transformed breast cancer cell lines compared to non-invasive controls was assessed using real-time quantitative PCR. Data represent mean ± SEM. Using unpaired, two-tailed t-test analysis. MCF-7-EMT n = 3; T47D-EMT n = 6; ***P < 0.0005; ****P < 0.0001 (B) Relative E-cadherin expression of mesenchymal transformed breast cancer cell lines compared to non-invasive controls was assessed using real-time quantitative PCR. Data represent mean ± SEM. Using unpaired, two-tailed t-test analysis. MCF-7-EMT n = 4; T47D-EMT n = 3; *P < 0.05; ****P < 0.0001 (C) Relative Vimentin expression of mesenchymal transformed breast cancer cell lines compared to non-invasive controls was assessed using real-time quantitative PCR. Data represent mean ± SEM. Using unpaired, two-tailed t-test analysis. MCF-7-EMT n = 5; T47D-EMT n = 3; *P < 0.05 (D) Relative Zeb1 expression of mesenchymal transformed breast cancer cell lines compared to non-invasive controls was assessed using real-time quantitative PCR. Data represent mean ± SEM. Using unpaired, two-tailed t-test analysis. MCF-7-EMT n = 4; T47D-EMT n = 3; *P < 0.05 (E) Relative SNAI1 expression of mesenchymal transformed breast cancer cell lines compared to non-invasive controls was assessed using real-time quantitative PCR. Data represent mean ± SEM. Using unpaired, two-tailed t-test analysis. MCF-7-EMT n = 4; T47D-EMT n = 5; ***P < 0.0005 (F) Relative SNAI2 expression of mesenchymal transformed breast cancer cell lines compared to non-invasive controls was assessed using real-time quantitative PCR. Data represent mean ± SEM. Using unpaired, two-tailed t-test analysis. MCF-7-EMT n = 3; T47D-EMT n = 4; *P < 0.05. [file Presentation_1.zip › S5.tif]
